# Normalized Multivariate Time Series Causality Analysis and Causal Graph Reconstruction

**DOI:** 10.3390/e23060679

**Published:** 2021-05-28

**Authors:** X. San Liang

**Affiliations:** 1 Nanjing Institute of Meteorology, Nanjing 210044, China; sanliang@courant.nyu.edu; 2 Shanghai Qizhi (Andrew C. Yao) Institute, Shanghai 200030, China; 3 China Institute for Advanced Study, Central University of Finance and Economics, Beijing 100081, China

**Keywords:** causal graph reconstruction, information flow, time series, synchronization

## Abstract

Causality analysis is an important problem lying at the heart of science, and is of particular importance in data science and machine learning. An endeavor during the past 16 years viewing causality as a real physical notion so as to formulate it from first principles, however, seems to have gone unnoticed. This study introduces to the community this line of work, with a long-due generalization of the information flow-based bivariate time series causal inference to multivariate series, based on the recent advance in theoretical development. The resulting formula is transparent, and can be implemented as a computationally very efficient algorithm for application. It can be normalized and tested for statistical significance. Different from the previous work along this line where only information flows are estimated, here an algorithm is also implemented to quantify the influence of a unit to itself. While this forms a challenge in some causal inferences, here it comes naturally, and hence the identification of self-loops in a causal graph is fulfilled automatically as the causalities along edges are inferred. To demonstrate the power of the approach, presented here are two applications in extreme situations. The first is a network of multivariate processes buried in heavy noises (with the noise-to-signal ratio exceeding 100), and the second a network with nearly synchronized chaotic oscillators. In both graphs, confounding processes exist. While it seems to be a challenge to reconstruct from given series these causal graphs, an easy application of the algorithm immediately reveals the desideratum. Particularly, the confounding processes have been accurately differentiated. Considering the surge of interest in the community, this study is very timely.

## 1. Introduction

Recent years have seen a surge of interest in causality analysis. The main thrust is the recognition of its increasing importance in machine learning and artificial intelligence, a milestone being the connection of the principle of independent causal mechanisms to semi-supervised learning by Schölkopf et al. [1]. Different methods have been proposed for inferring the causality from data, in addition to the classical ones such as Granger causality testing. While traditionally causal inference has been categorized as a subject in statistics, and now also a subject in computer science, it merits mentioning that, during recent decades, contributions from different disciplines have augmented the subject and significant advances have been made ever since. Early efforts since Clive Granger and Judea Pearl (cf. [2] include, for example, Spirtes and Glymour (1991) [3], Schreiber (2000) [4], Paluš et al. (2001) [5], and Liang and Kleeman (2005) [6]. Recently, due to the rush in artificial intelligence, publications have been growing rapidly, among which are Zhang and Spirtes (2008) [7], Maathuis et al. (2009) [8], Pompe and Runge (2011) [9], Janzing et al. (2012) [10], Sugihara et al. (2012) [11], Schölkopf et al. (2012) [1], Sun and Bollt (2014) [12], Peters et al. (2017) [13], to name but a few; see [13] and [14] for more references.

Although causality has long been investigated ever since Granger [15], thanks to the systematic works by Pearl (e.g., [2]) and others, its “mathematization is a relatively recent development,” said Peters, Janzing and Schökopf (2017) [13]. On the other hand, Liang (2016) [16] argued that it is actually “a real physical notion that can be derived *ab initio*.” Despite the current rush, this latter line of work, which starts some 16 years ago, seems to have gone almost unnoticed. It can be traced back to a discovery of two-dimensional (2D) information flow in 2005 by Liang and Kleeman [6]. With the later efforts of, e.g., Liang (2008) [17] and Liang (2014) [18], a very easy method for bivariate time series causality analysis has been established, validated, and applied successfully to real world problems in different disciplines. More details can be found below in Section 2. Recently, the whole formalism has been put on a rigorous footing [16]; explicit formulas for multidimensional information flow have been obtained in a closed form with both deterministic and stochastic systems.

The multivariate time series causality analysis, however, has not been established since Liang (2016)’s comprehensive work [16]. Considering the enormous interest in this field, we are henceforth intented to fill the gap. The purpose of this study is hence two-fold: (1) Implement Liang (2016)’s theory into the long-due multivariate time series causality analysis; (2) along with the implementation present a brief introduction of this line of work.

The remaining of the paper is organized as follows. In Section 2, we first establish the framework, and then take a stroll through the theory of information flow and the information flow-based bivariate time series causality analysis. Section 3 presents an estimate of the information flow rates among multivariate time series, and their significance tests. These information flows can be normalized to reveal the impact of the role in question (Section 4). In order to test the power of the method, in Section 5, it is applied to infer the causal graphs with two extreme processes, one being a network with heavy noise (noise-to-signal ratio exceeding 100), another being a network of almost synchronized chaotic oscillators. Section 6 closes the paper with a brief summary of the study.

## 2. An Overview of the Theory of Information Flow-Based Causality Analysis

### 2.1. Directed Graph, Uncertainty Propagation, and Causality

In this framework, causal inference is based on information flow (rather than the other way around), which has been recognized as a real physical notion that can be put on a rigorous footing (see Liang, 2016). Consider a graph (V,E), where *V* and *E* are the sets of vertexes and edges, and the structural causal model on the graph, $\left(C,P_{N}\right)$, where C is a collection of *d* structural assignments $X_{i}=F_{i}\left(\mathrm{PA}(X_{i}),\epsilon_{i}\right)$, i=1,…,d, $\mathrm{PA}\left(X_{i}\right)\subseteq \left\{X_{\backslash \;i}\right\}=X_{1},\ldots ,X_{d}\}\backslash \left\{X_{i}\right\}$ indicating the parents or direct causes of $X_{i}$, and $P_{N}$ being a joint distribution over the noise variables [2]. The basic idea is that this can be recast within the framework of dynamical systems, and that the causal inference problem can be carried forth to that between the coordinates in a dynamical system. This is how Liang and Kleeman (2005) [6] originally conceptualized the problem. Recently, it has also been realized by Røysland (2012) [19], Mooij et al. (2013) [20] and Mogensen et al. (2018) [21].

In physics there is a notion called information flow, which can be readily cast within the dynamical system framework. As Shannon entropy (simply “entropy” hereafter) is by interpretation “self information”, it is natural to measure it with the propagation of entropy or uncertainty, from one component to another. (Other entropies may provide alternative choices. Particularly, a generalized permutation entropy is referred to [22].) In this light, we have the following definition:

**Definition** **1.** *In a dynamical system $\left(\Omega ,\Phi_{t}\right)$ on the d-dimensional phase space *Ω*, where $\Phi_{t}$ may be a continuous-time flow ($t\in {\mathbb{R}}^{+}$) or discrete-time mapping $t\in Z^{+}$), the information flow from a component/coordinate $X_{j}$ to another component/coordinate $X_{i}$, written $T_{j\to i}$, is defined as the contribution of entropy (uncertainty) from $X_{j}$ per unit time ($t\in {\mathbb{R}}^{+}$) or per step ($t\in Z^{+}$) in increasing the marginal entropy of $X_{i}$.*

With information flow, causality can be defined, and, moreover, quantitatively defined:

**Definition** **2.** *$X_{j}$ is causal to $X_{i}$ iff $T_{j\to i}\neq 0$. The magnitude of the causality from $X_{j}$ to $X_{i}$ is measured by $|T_{j\to i}|$.*

By evaluating the information flow within a dynamical system, the underlying causal graph is henceforth determined.

For this study, we consider only the continuous flow case. The vector field that forms the structural assignments is hence differentiable. Further, we assume a Wiener process for the noise (white noise). Note that some of these assumptions can be easily relaxed, and the generalization is straightforward. However, that is outside the scope of this study.

### 2.2. A Brief Stroll through the Theory and Recent Advances

This line of work begins with Liang and Kleeman (2005) [6] within the framework of 2D deterministic systems. Originally, it is based on a heuristic argument, but later on it is rigorized. Its generalization to multidimensional and stochastic systems, however, has not been fulfilled until the recent theoretical work by Liang (2016) [16]. The following is just a brief review.

We begin by stating an observational fact about causality:

*If the evolution of an event, say, $X_{1}$, is independent of another one, $X_{2}$, then the information flow from $X_{2}$ to $X_{1}$ is zero.*

Since it is the only quantitatively stated fact about causality, all previous empirical/half-empirical causality formalisms have attempted to verify it in applications. For this reason, it has been referred to as the principle of nil causality (e.g., [16]). We will soon see below that, within the information flow framework, this principle turns out to be a proven theorem.

Consider a *d*-dimensional continuous-time stochastic system for $X=(X_{1},\ldots ,X_{d})$

(1) $$\begin{array}{c} dX=F(X,t)dt+B(X,t)dW, \end{array}$$

where $F=(F_{1},\ldots ,F_{d})$ may be arbitrary nonlinear functions of X and *t*, W is a vector of standard Wiener processes, and $B=(b_{ij})$ is the matrix of perturbation amplitudes, which may also be any functions of X and *t*. Assume that F and B are both differentiable with respect to X and *t*. We then have the following theorem [16]:

**Theorem** **1.** *For the system (1), the rate of information flowing from $X_{j}$ to $X_{i}$ (in nats per unit time) is*

(2) $$\begin{array}{ccc} T_{j\to i} & = & -E\left[\frac{1}{\rho_{i}}\int_{{\mathbb{R}}^{d-2}}\frac{\partial (F_{i}\rho_{\backslash \;j})}{\partial x_{i}}dx_{\backslash \;i\backslash \;j}\right]+\\ & & \;\frac{1}{2}E\left[\frac{1}{\rho_{i}}\int_{{\mathbb{R}}^{d-2}}\frac{\partial^{2}\left(g_{ii}\rho_{\backslash \;j}\right)}{\partial x_{i}^{2}}dx_{\backslash \;i\backslash \;j}\right],\\ & = & -\int_{{\mathbb{R}}^{d}}\rho_{j|i}\left(x_{j}|x_{i}\right)\frac{\partial (F_{i}\rho_{\backslash \;j})}{\partial x_{i}}dx+\\ & & \;\frac{1}{2}\int_{{\mathbb{R}}^{d}}\rho_{j|i}\left(x_{\backslash \;j}|x_{i}\right)\frac{\partial^{2}\left(g_{ii}\rho_{\backslash \;j}\right)}{\partial x_{i}^{2}}dx, \end{array}$$

*where $dx_{\backslash \;i\backslash \;j}$ signifies $dx_{1}\ldots dx_{i-1}dx_{i+1}\ldots dx_{j-1}dx_{j+1}\ldots dx_{n}$, E stands for mathematical expectation, $g_{ii}=\sum_{k=1}^{n}b_{ik}b_{ik}$, $\rho_{i}=\rho_{i}\left(x_{i}\right)$ is the marginal probability density function (pdf) of $X_{i}$, $\rho_{j|i}$ is the pdf of $X_{j}$ conditioned on $X_{i}$, and $\rho_{\backslash \;j}=\int_{\mathbb{R}}\rho \left(x\right)dx_{j}$.*

For discrete-time mappings, the information flow is in a more complicated form; see [16].

**Corollary** **1.** *When d=2,*

(3) $$\begin{array}{c} T_{2\to 1}=-E\left[\frac{1}{\rho_{1}}\frac{\partial (F_{1}\rho_{1})}{\partial x_{1}}\right]+\frac{1}{2}E\left[\frac{1}{\rho_{1}}\frac{\partial^{2}(g_{11}\rho_{1}}{\partial x_{1}^{2}})\right]. \end{array}$$

This is the early result of Liang (2008) [17] on which the bivariate causality analysis is based; see Theorem 5 below.

There is a nice property for the above information flow:

**Theorem** **2.** *If in (1) neither $F_{1}$ nor $g_{11}$ depends on $X_{2}$, then $T_{2\to 1}=0$.*

Note this is precisely the principle of nil causality. Remarkably, here it appears as a proven theorem, while the classical ansatz-like formalisms attempt to verify it in applications.

Moreover, it has been established that [23]:

**Theorem** **3.** *$T_{2\to 1}$ is invariant under arbitrary nonlinear transformation of $\left(X_{3},X_{4},\ldots ,X_{d}\right)$.*

This is a very important result, as we will see soon in the causal graph reconstruction. On the other hand, this tells that the obtained information flow should be an intrinsic property in physical world.

For linear systems, the information flow can be greatly simplified.

**Theorem** **4.** *In (1), if F(X)=f+AX, and B is a constant matrix, then*

(4) $$\begin{array}{c} T_{j\to i}=a_{ij}\frac{\sigma_{ij}}{\sigma_{ii}}, \end{array}$$

*where $a_{ij}$ is the ${\left(i,j\right)}^{th}$ entry of A, and $\sigma_{ij}$ the population covariance between $X_{i}$ and $X_{j}$.*

Observe that, if $\sigma_{ij}=0$, then $T_{j\to i}=0$; but if $T_{j\to i}=0$, $\sigma_{ij}$ does not necessarily vanish. Contrapositively, this means that correlation does not mean causation. We hence have the following corollary:

**Corollary** **2.** *In the linear sense, causation implies correlation, but correlation does not imply causation.*

This explicit mathematical expression hence provides a solution to the long-standing debate ever since George Berkeley (1710) [24] over correlation versus causation. Note, however, this is for linear systems only. For nonlinear systems, the existence of such a relation, and, if existing, how it is like, are yet to be explored. Nonetheless, as proved in [25], this relation indeed holds for some counter-examples in terms of normalized information flow (see Section 4 below).

In the case with only two time series (no dynamical system is given), we have the following result [18]:

**Theorem** **5.** *Given two time series $X_{1}$ and $X_{2}$, under the assumption of a linear model with additive noise, the maximum-likelihood estimator (mle) of (3) is*

(5) $$\begin{array}{c} {\hat{T}}_{2\to 1}=\frac{C_{11}C_{12}C_{2,d1}-C_{12}^{2}C_{1,d1}}{C_{11}^{2}C_{22}-C_{11}C_{12}^{2}}, \end{array}$$

*where $C_{ij}$ is the sample covariance between $X_{i}$ and $X_{j}$, and $C_{i,dj}$ is the sample covariance between $X_{i}$ and a series derived from $X_{j}$ using the Euler forward differencing scheme: ${\dot{X}}_{j,n}=\left(X_{j,n+k}-X_{j,n}\right)/\left(k\Delta t\right)$, with k≥1 some integer.*

Equation (5) is rather concise in form; it only involves the common statistics, i.e., sample covariances. In other words, a combination of some sample covariances will give a quantitative measure of the causality between the time series. This makes causality analysis, which otherwise would be complicated with the classical empirical/half-empirical methods, very easy. Nonetheless, note that Equation (5) cannot replace (3); it is just the mle of the latter. A statistical significance test must be performed before a causal inference is made based on the computed ${\hat{T}}_{2\to 1}$. For details, refer to [18].

The above formalism has been validated with many benchmark systems such as baker transformation, Hénon map, Kaplan–Yorke map, Rössler system (see [16]), to name a few. Particularly, the concise Equation (5) has been validated with problems where traditional approaches fail. An example is the mysterious anticipatory system problem discovered by Hahs and Pethel [26], which with (5) is successfully fixed in an easy way.

The formalism has been successfully applied to the studies of many real world problems, among them are the El Niño-Indian Ocean Dipole relation [18], global climate change [27], soil moisture–precipitation interaction [28], glaciology [29], and neuroscience problems [30], to name a few. Here, we particularly want to mention the study by Stips et al. [27], who, through examining with (5) the causality between the CO${}_{2}$ index and the surface air temperature, identified a reversing causal relation with time scale. They found, during the past century, indeed CO${}_{2}$ emission drives the recent global warming; the causal relation is one-way, i.e., from CO${}_{2}$ to global mean atmosphere temperature. Moreover, they were able to find how the causality is distributed over the globe, thanks to the quantitative nature of (5). However, on a time scale of 1000 years or over, the causality is completely reversed; that is to say, on a paleoclimate scale, it is global warming that drives the CO${}_{2}$ concentration to rise.

## 3. Information Flow among Time Series and Algorithm for Multivariate Causal Inference

We now estimate (2), given observations of the *d* components, in order to arrive at a practically easy-to-use formula for causal inference. As mentioned in Section 1, this has not been done yet; the available estimator (5) is for (3). Here, we only consider time series, but it can be easily extended to other forms of data. We further assume the series are stationary and equi-distanced. Without loss of generality, it suffices to examine $T_{2\to 1}$.

As in the bivariate case considered in [18], we estimate the linear version (4). We hence examine a linear stochastic differential equation

(6) $$\begin{array}{c} dX=f+AXdt+BdW, \end{array}$$

where f is a constant vector, and $A=(a_{ij})$ and $B=(b_{ij})$ are constant matrices. Initially, if X obeys a Gaussian distribution, then it is a Gaussian for ever, i.e., X∼N(μ,Σ), with $\mu ={\left(\mu_{1},\ldots ,\mu_{d}\right)}^{T}$ and $\Sigma =(\sigma_{ij})$ being the mean vector and covariance matrix, respectively. Hence, $X_{1}∼N\left(\mu_{1},\sigma_{11}\right)$.

The above results need to be estimated if what we are given are just *d* time series. That is to say, what we know is a single realization of some unknown system, which, if known, can produce infinitely many realizations. We use maximum-likelihood estimation (e.g., [31]) to achieve the goal. The procedure follows that of [18], which for easy reference, we briefly summarize here. As established before, a further assumption that B is diagonal, i.e., $b_{ij}=0$, for i≠j, and hence $g_{11}=b_{11}^{2}$, will greatly simplify the problem, while in practice, this is quite reasonable.

Suppose that the series are equal-distanced with a time stepsize Δt, and let *N* be the sample size. Consider an interval [nΔt,(n+1)Δt], and let the transition pdf be $\rho (X_{n+1}|X_{n};\theta )$, where θ stands for the vector of parameters to be estimated. So, the log likelihood is

$$\begin{array}{c} \ell_{N}\left(\theta \right)=\sum_{n=1}^{N}\log\rho \left(X_{n+1}|X_{n};\theta \right)+\log\rho \left(X_{1}\right). \end{array}$$

As *N* is usually large, the term $\rho (X_{1})$ can be dropped without causing much error. The transition pdf is, with the Euler–Bernstein approximation (see [18]),

$$\begin{array}{ccc} & & \rho \left(X_{n+1}=x_{n+1}|X_{n}=x_{n}\right)={\left[{\left(2\pi \right)}^{d}\det\left(BB^{T}\Delta t\right)\right]}^{-1/2}\\ & & \;\times e^{-\frac{1}{2}{\left(x_{n+1}-x_{n}-F\Delta t\right)}^{T}{\left(BB^{T}\Delta t\right)}^{-1}\left(x_{n+1}-x_{n}-F\Delta t\right)}, \end{array}$$

where F=f+AX. This results in a log likelihood functional

$$\begin{array}{c} \ell_{N}\left(f,A,B\right)=\mathrm{const}-\frac{N}{2}\log\prod_{i}g_{ii}-\frac{\Delta t}{2}\left(\frac{1}{\sum_{i=1}^{d}g_{ii}}\sum_{n=1}^{N}R_{i,n}^{2}\right), \end{array}$$

where

$$\begin{array}{c} R_{i,n}={\dot{X}}_{i,n}-\left(f_{i}+\sum_{j=1}^{d}a_{ij}X_{j,n}\right),\;i=1,2,\ldots ,d \end{array}$$

and ${\dot{X}}_{i}=\left\{{\dot{X}}_{i,n}\right\}$ is the Euler forward differencing approximation of $\frac{dX_{i}}{dt}$:

(7) $$\begin{array}{c} {\dot{X}}_{i,n}=\frac{X_{i,n+k}-X_{i,n}}{k\Delta t}, \end{array}$$

with k≥1. Usually, k=1 should be used to ensure accuracy, but in some cases of deterministic chaos and the sampling is at the highest resolution, one needs to choose k=2. Maximizing $\ell_{N}$, it is easy to find that the maximizer $\left({\hat{f}}_{1},{\hat{a}}_{11},\ldots {\hat{a}}_{1d}\right)$ satisfies the following algebraic equation:

(8) $$\begin{array}{c} \left[\begin{array}{cccc} 1 & \bar{X_{1}} & \ldots & \bar{X_{d}}\\ \bar{X_{1}} & \bar{X_{1}^{2}} & \ldots & \bar{X_{1}X_{d}}\\ \vdots & \vdots & \ddots & \vdots \\ \bar{X_{d}} & \bar{X_{1}X_{d}} & \ldots & \bar{X_{d}^{2}} \end{array}\right]\left(\begin{array}{c} {\hat{f}}_{1}\\ {\hat{a}}_{11}\\ \vdots \\ {\hat{a}}_{1d} \end{array}\right)=\left(\begin{array}{c} \bar{{\dot{X}}_{1}}\\ \bar{X_{1}{\dot{X}}_{1}}\\ \vdots \\ \bar{X_{d}{\dot{X}}_{1}} \end{array}\right) \end{array}$$

where the overline signifies sample mean. After some algebraic manipulations as that in [18], this yields the maximum-likelihood estimators (mle):

(9) $$\begin{array}{ccc} & & {\hat{a}}_{1i}=\frac{1}{\det C}\sum_{j=1}^{d}\Delta_{ij}C_{j,d1} \end{array}$$

(10) $$\begin{array}{ccc} & & {\hat{g}}_{11}=\frac{Q_{N,1}\Delta t}{N}, \end{array}$$

(11) $$\begin{array}{ccc} & & {\hat{f}}_{1}=\bar{{\dot{X}}_{1}}-\sum_{i=1}^{d}{\hat{a}}_{1i}{\bar{X}}_{i}, \end{array}$$

where

(12) $$\begin{array}{ccc} & & C_{ij}=\bar{\left(X_{i}-{\bar{X}}_{i}\right)\left(X_{j}-{\bar{X}}_{j}\right)}, \end{array}$$

(13) $$\begin{array}{ccc} & & C_{i,dj}=\bar{\left(X_{i}-{\bar{X}}_{i}\right)\left({\dot{X}}_{j}-\bar{{\dot{X}}_{j}}\right)}, \end{array}$$

are the sample covariances, $\Delta_{ij}$ the cofactors of the matrix $C=(C_{ij})$, and

$$\begin{array}{ccc} Q_{N,1} & = & \sum_{n=1}^{N}{\left[{\dot{X}}_{1,n}-\left({\hat{f}}_{1}+\sum_{j=1}^{d}{\hat{a}}_{1j}X_{j,n}\right)\right]}^{2}\\ & = & \sum_{n=1}^{N}{\left[\left({\dot{X}}_{1,n}-\bar{{\dot{X}}_{1}}\right)-\sum_{i=1}^{d}{\hat{a}}_{1i}\left(X_{i,n}-{\bar{X}}_{i}\right)\right]}^{2}\\ & = & N(C_{d1,d1}-2\sum_{i=1}^{d}{\hat{a}}_{1i}C_{d1,i}+\sum_{i=1}^{d}\sum_{j=1}^{d}{\hat{a}}_{1i}{\hat{a}}_{1j}C_{ij}. \end{array}$$

By (4), this yields an estimator of the information flow from $X_{2}$ to $X_{1}$:

(14) $$\begin{array}{c} {\hat{T}}_{2\to 1}=\frac{1}{\det C}\cdot \sum_{j=1}^{d}\Delta_{2j}C_{j,d1}\cdot \frac{C_{12}}{C_{11}}, \end{array}$$

where $C_{j,d1}$ is the sample covariance between $X_{j}$ and the derived series ${\dot{X}}_{1}$ as computed by (7). When d=2, it is easy to show that this is reduced to (5), the 2D estimator as obtained in [18].

Information flow concerns the influence from one element to another element, i.e., the causal relation between different elements. A relation can also contain two identical elements; this corresponds to a self-loop in a graph. Historically, before establishing the information flow from, say $X_{2}$, to another component, say $X_{1}$, the contribution of the change of marginal entropy of $X_{1}$ by itself is first established. This contribution, denoted by $dH_{1}^{*}/dt$, proves to be $E(\frac{\partial F_{1}}{\partial x_{1}}$ (cf. [16]). As we can see from above, besides the estimator of information flow, in this study, we actually have also estimated $dH_{1}^{*}/dt$, i.e., the influence of a component (here $X_{1}$) on itself.

**Theorem** **6.** Under a linear assumption, the maximum-likelihood estimator of $dH_{1}^{*}/dt$ is

(15) $$\begin{array}{c} \hat{\left(\frac{dH_{1}^{*}}{dt}\right)}=\frac{1}{\det C}\cdot \sum_{j=1}^{d}\Delta_{1j}C_{j,d1}. \end{array}$$

**Proof.**  Since $dH_{1}^{*}/dt=E\left(\frac{\partial F_{1}}{\partial x_{1}}\right)$, which is $a_{11}$ in this case. The mle hence follows. □

This supplies information not seen in previous causality analysis along this line. As will be clear soon, this helps identify self loops in a causal graph.

Statistical significance tests can be performed for (14) and (15). When *N* is large, they are approximately normally distributed around their true values with variances ${\left(\frac{C_{12}}{C_{11}}\right)}^{2}{\hat{\sigma }}_{a_{12}}^{2}$ and ${\hat{\sigma }}_{a_{11}}^{2}$, respectively, thanks to the mle property. Here, ${\hat{\sigma }}_{a_{12}}^{2}$ and ${\hat{\sigma }}_{a_{11}}^{2}$ are determined as follows (e.g., [31]). Denote $\theta =(f_{1},a_{11},a_{12},\ldots ,a_{1d},b_{1})$. Compute

$$\begin{array}{c} I_{ij}=-\frac{1}{N}\sum_{n=1}^{N}\frac{\partial^{2}\log\rho \left(X_{n+1}|X_{n};\;\hat{\theta }\right)}{\partial \theta_{i}\partial \theta_{j}} \end{array}$$

to form a (d+2)×(d+2) matrix I, namely, the Fisher information matrix. The inverse ${\left(NI\right)}^{-1}$ is the covariance matrix of $\hat{\theta }$, within which are ${\hat{\sigma }}_{a_{12}}^{2}$ and ${\hat{\sigma }}_{a_{11}}^{2}$. Given a significance level, the confidence interval can be found accordingly.

From the above, an algorithm for causal inference hence can be implemented, as shown in Algorithm 1.
**Algorithm 1:** Quantitative causal inference **Input**    : *d* time series
 **Output**: a DG G=(V,E), and IFs along edges initialize G such that all vertexes are isolated;
 set a significance level α;
 **for each** (i,j)∈V×V **do**
   compute ${\hat{T}}_{i\to j}$ by (14); 
   **if** ${\hat{T}}_{i\to j}$ is significant at level α
**then**
    add i→j to G; 
    record ${\hat{T}}_{i\to j}$;    **end**
 **end**
 return G, together with the IFs ${\hat{T}}_{i\to j}$


## 4. Normalization of the Causality among Multivariate Time Series

In many problems, just an assertion whether a causality exists is not enough; we need to know how important it is. This raises an issue of normalization. The normalization of information flow is by no means as trivial as it seemingly looks. Quite different from the case of covariance vs. correlation coefficient, no such relation as Cauchy–Schwartz inequality exists. Liang [32] listed some difficulties in the problem, and so far this is still an area of research. Hereafter, we follow [32] to propose the normalizer for (14).

The basic idea is that the normalizer for $T_{2\to 1}$ should be related to $dH_{1}/dt$, as the former is by derivation a part of the contribution to the latter. However, $dH_{1}/dt$ itself cannot be the normalizer, since many terms tend to cancel; sometimes $dH_{1}/dt$ may even completely vanish, just as in the Hénon map case. We now write out the estimator of $dH_{1}/dt$ and see how the problem can be fixed.

By [16], the time rate of change of the marginal entropy of $X_{1}$ is

(16) $$\begin{array}{c} \frac{dH_{1}}{dt}=-E\left(F_{1}\frac{\partial \log\rho_{1}}{\partial x_{1}}\right)-\frac{1}{2}E\left(g_{11}\frac{\partial^{2}\log\rho_{1}}{\partial x_{1}^{2}}\right). \end{array}$$

In this linear case,

(17) $$\begin{array}{ccc} \frac{dH_{1}}{dt} & = & -E\left(\sum_{j=1}^{d}a_{1j}X_{j}\frac{\partial \log\rho_{1}}{\partial x_{1}}\right)-\frac{1}{2}E\left(g_{11}\frac{\partial^{2}\log\rho_{1}}{\partial x_{1}^{2}}\right)\\ & = & E\left(\frac{X_{1}-\mu_{1}}{\sigma_{11}}\sum_{j}a_{1j}X_{j}\right)+\frac{1}{2}\frac{g_{11}}{\sigma_{11}}\\ & = & a_{11}+\sum_{j=2}^{d}T_{j\to 1}+\frac{1}{2}\frac{g_{11}}{\sigma_{11}}. \end{array}$$

The first term is $dH_{1}^{*}/dt$, i.e., the contribution from itself, and the last term is the effect of noise, written $dH_{1}^{noise}/dt$. The remaining parts are the information flows to $X_{1}$, just as expected. We may hence propose a normalizer as follows:

(18) $$\begin{array}{c} Z\equiv |\frac{dH_{1}^{*}}{dt}|+\sum_{j=2}^{d}|T_{j\to 1}\left|+\right|\frac{dH_{1}^{noise}}{dt}|. \end{array}$$

Hence, the normalized information flow from $X_{2}$ to $X_{1}$ is:

(19) $$\begin{array}{c} \tau_{2\to 1}=\frac{T_{2\to 1}}{Z}. \end{array}$$

Clearly, $\tau_{2\to 1}$ lies on [−1,1]. So, when $|\tau_{2\to 1}|$ is 100%, $X_{2}$ has the maximal impact on $X_{1}$.

Note that ${\frac{dH}{dt}}_{1}^{noise}=g_{11}/\left(2\sigma_{11}\right)$, where $g_{11}=\sum_{j=1}^{d}b_{1j}^{2}$ is always positive. That is to say, noise always contributes to increase the marginal entropy of $X_{1}$, agreeing with our common sense. Obviously, this term is related to the noise-to-signal ratio.

By the results in Section 3, *Z* can be estimated as

(20) $$\begin{array}{c} \hat{Z}=|\hat{\left(\frac{dH_{1}^{*}}{dt}\right)}|+\sum_{j=2}^{d}|{\hat{T}}_{j\to 1}\left|+\right|\hat{\left(\frac{dH_{1}^{noise}}{dt}\right)}|. \end{array}$$

where $\hat{\left(\frac{dH_{1}^{noise}}{dt}\right)}=\frac{1}{2}\frac{{\hat{g}}_{11}}{C_{11}}$, and ${\hat{g}}_{11}$, $\hat{\left(\frac{dH_{1}^{*}}{dt}\right)}$ and ${\hat{T}}_{2\to 1}$ are evaluated using (10), (14) and (15), respectively.

## 5. Application to Causal Graph Reconstruction

### 5.1. A Noisy Causal Network from Autoregressive Processes

Consider the series generated from a *d*-dimensional vector autoregressive (VAR) process:

(21) $$\begin{array}{c} X(n+1)=\alpha +AX(n)+Be(n+1) \end{array}$$

where $X={\left(X_{1},\ldots ,X_{d}\right)}^{T}$, $A={\left(a_{ij}\right)}_{d\times d}$, $e={\left(e_{1},\ldots ,e_{d}\right)}^{T}$, and B is a diagonal matrix with diagonal entries $b_{ii}$, i=1,…,d. Here, the errors $e_{i}∼N\left(0,1\right)$ are independent, and $b_{i}$ are the amplitudes of stochastic perturbation. Let

$$\begin{array}{ccc} & & A=\left(\begin{array}{cccccc} 0 & 0 & -0.6 & 0 & 0 & 0\\ -0.5 & 0 & 0 & 0 & 0 & 0.8\\ 0 & 0.7 & 0 & 0 & 0 & 0\\ 0 & 0 & 0 & 0.7 & 0.4 & 0\\ 0 & 0 & 0 & 0.2 & 0 & 0.7\\ 0 & 0 & 0 & 0 & 0 & -0.5 \end{array}\right),\\ & & \alpha ={\left(0.1,0.7,0.5,0.2,0.8,0.3\right)}^{T}, \end{array}$$

**Figure 1:**
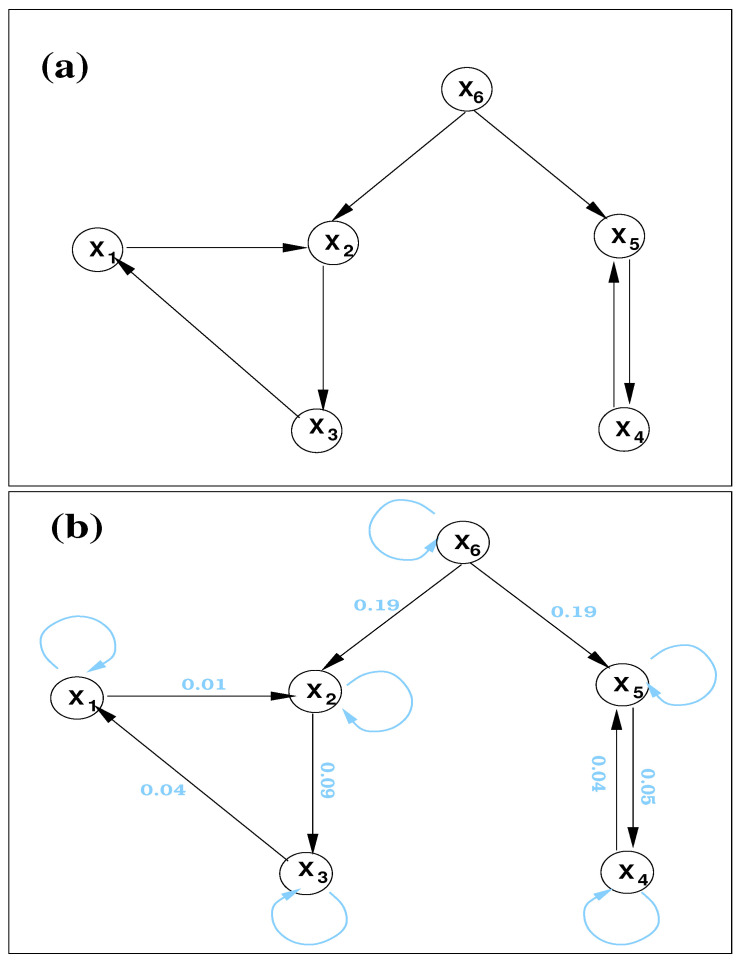
(**a**) A schematic of the directed network generated with the vector autoregressive processes (21). (**b**) The directed graph reconstructed from the six time series. Overlaid numbers are the respective significant information flows (in nats per time step); also overlaid are the inferred self loops or trivial cycles of length 1 (in light blue).

The formed network is as shown in Figure 1a. So, by design, we have two directed cycles ($X_{1}$,$X_{2}$,$X_{3}$) and ($X_{4}$,$X_{5}$). The former is of length 3, while the latter are parallel edges. These cycles are driven by a common cause or confounder $X_{6}$. Since no diagonal entries of A is 1, all nodes are self loops (trivial cycles of length 1). The resulting autocorrelation is believed to be a challenge in causal inferences for some techniques. This and the confoundingness of $X_{6}$, have been two major issues for many causal inference methods.

First, consider the case $b_{ii}=1$. Accordingly, six series of 10,000 steps are generated (randomly initialized).

By computation, the information flow rates are (only absolute values are shown), if arranged in a matrix form such that the ${\left(i,j\right)}^{\mathrm{th}}$ entry indicates $|T_{i\to j}|$, then the absolute information flow rates are

$$\begin{array}{c} \left(\begin{array}{cccccc} \backslash & 0.01\; & 0.00\; & 0.00\; & 0.00\; & 0.00\\ 0.00\; & \backslash & 0.09 & 0.00\; & 0.00 & 0.00\\ 0.05 & 0.00 & \backslash & 0.00 & 0.00 & 0.00\\ 0.00 & 0.00 & 0.00 & \backslash & 0.04 & 0.00\\ 0.00 & 0.00 & 0.00 & 0.05 & \backslash & 0.00\\ 0.00 & 0.19 & 0.00 & 0.00 & 0.18 & \backslash \end{array}\right), \end{array}$$

So, the only significant information flows (numbers in bold) are $T_{1\to 2}$, $T_{2\to 3}$, $T_{3\to 1}$, $T_{4\to 5}$, $T_{5\to 4}$, $T_{6\to 2}$, $T_{6\to 5}$, as indicated in Figure 1b. (At a 90% confidence level, the maximal error is 0.005, so all these values are significant.) This is precisely the same as designed. So, the causal graph is accurately reconstructed. Also, by (15) $|dH_{1}^{*}/dt\left|,\ldots ,\right|dH_{6}^{*}/dt|$ can be computed. They are 1.00±0.01, 1.01±0.01, 1.01±0.01, 0.30±0.01, 1.00±0.01, 1.49±0.02, where the errors at a 90% confidence level are shown. So, here all the nodes are self loops (trivial cycles of length 1).

It should be particularly pointed out that the confoundingness of $X_{6}$ does not make an issue here. As shown in Figure 1, there is no significant information flow between $X_{2}$ and $X_{5}$; in other words, they are not directly causal to each other. Nor are $X_{3}$ and $X_{4}$. This is actually not a surprise; it is a corollary of the principle of nil causality, as proved before (see Theorem 2). Considering the difficulty of this problem, the performance of this concise Formula (14) is remarkable.

The above information flows can be normalized to understand the impact of one unit on another. For example, $|\tau_{6\to 2}|=13.2\%$, $|\tau_{6\to 5}=12.5\%$. For another example, in the cycle ($X_{4}$, $X_{5}$), the relative information flows are $\tau_{4\to 5}=2.4\%$, $\tau_{5\to 4}=8.8\%$, in contrast to the almost identical absolute information flows. This is understandable: though $T_{5\to 4}$ is comparable to $T_{4\to 5}$, the parts contributing to $dH_{5}/dt$ are different from that to $dH_{4}/dt$, and thus they may have different weights.

Now, consider an extreme case when the signals are buried within heavy noise. Let, $b_{ii}=100$, and repeat the above steps. The results are, remarkably, almost the same. So, the Formula (14) is very robust in the presence of noise.

If the time series is short, the performance is still satisfactory. For example, if it has only 500 data points, the above case with heavy noise ($b_{ii}=100$) results in the following matrix of information flow rates:

$$\begin{array}{c} \left(\begin{array}{cccccc} \backslash & 0.02\; & 0.00\; & 0.00\; & 0.00\; & 0.00\\ 0.00\; & \backslash & 0.13 & 0.00\; & 0.01 & 0.01\\ 0.04 & 0.01 & \backslash & 0.00 & 0.00 & 0.00\\ 0.00 & 0.00 & 0.00 & \backslash & 0.07 & 0.00\\ 0.01 & 0.00 & 0.00 & 0.06 & \backslash & 0.00\\ 0.00 & 0.17 & 0.00 & 0.00 & 0.19 & \backslash \end{array}\right), \end{array}$$

with the corresponding errors at the 90% confidence level being:

$$\begin{array}{c} \left(\begin{array}{cccccc} \backslash & 0.00\;\; & 0.00\;\; & 0.00\;\; & 0.00\;\; & 0.01\\ 0.00\;\; & \backslash & 0.01 & 0.00\; & 0.02 & 0.02\\ 0.00 & 0.01 & \backslash & 0.00 & 0.01 & 0.01\\ 0.00 & 0.00 & 0.01 & \backslash & 0.01 & 0.00\\ 0.01 & 0.00 & 0.00 & 0.06 & \backslash & 0.02\\ 0.01 & 0.01 & 0.01 & 0.00 & 0.02 & \backslash \end{array}\right). \end{array}$$

So, the significant (at the 90% level) information flows are still those as shown in bold face.

Note we do not mean to compete with the classical method(s) in this application. Granger causality testing, for example, works well here. Nonetheless, the simplicity of the Formula (14) and the algorithm has greatly increased the performance of computation. On MATLAB, (14) is by test more than 100 times faster than the embedded matlab function gctest.

### 5.2. A Network of Nearly Synchronized Chaotic Series

Now, consider the following causal graph made of Rössler oscillators *X*, *Y* and *Z*, where *X* is a confounder. A Rössler oscillator has three components, so the system actually has a dimension of 9.

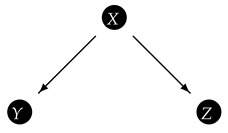



We use for this purpose the coupled system investigated by Palu$\overset{ˇ}{s}$ et al. [33]. The 9 series are generated through the following Rössler systems

$$\begin{array}{ccc} & & \left\{\begin{array}{c} dx_{1}/dt=-\omega_{1}x_{2}\left(t\right)-x_{3}\left(t\right),\\ dx_{2}/dt=\omega_{1}x_{1}\left(t\right)+0.15x_{2}\left(t\right),\\ dx_{3}/dt=0.2+x_{3}\left(t\right)\left[x_{1}\left(t\right)-10\right], \end{array}\right.\\ & & \left\{\begin{array}{c} dy_{1}/dt=-\omega_{2}y_{2}\left(t\right)-y_{3}\left(t\right)+\epsilon \left[x_{1}\left(t\right)-y_{1}\left(t\right)\right],\\ dy_{2}/dt=\omega_{2}y_{1}\left(t\right)+0.15y_{2}\left(t\right),\\ dy_{3}/dt=0.2+y_{3}\left(t\right)\left[y_{1}\left(t\right)-10\right], \end{array}\right.\\ & & \left\{\begin{array}{c} dz_{1}/dt=-\omega_{3}z_{2}\left(t\right)-z_{3}\left(t\right)+\epsilon \left[x_{1}\left(t\right)-z_{1}\left(t\right)\right],\\ dz_{2}/dt=\omega_{3}z_{1}\left(t\right)+0.15z_{2}\left(t\right),\\ dz_{3}/dt=0.2+z_{3}\left(t\right)\left[z_{1}\left(t\right)-10\right]. \end{array}\right. \end{array}$$

Clearly, the first is the driving or “master” system, while the latter two are slaves which are not directly connected. We hence use them to define *X*, *Y* and *Z*. This system is exactly the same as the one studied in [33], except for the addition of another subsystem, *Z*. The parameters are also chosen to be the same as theirs, $\omega_{1}=1.015$ and $\omega_{2}=0.985$, but with an additional one, $\omega_{3}=0.95$. As can be seen, *X* is coupled with *Y* and *Z* through the first component, and the coupling is one-way, i.e., from *X* to *Y* and from *X* to *Z*. The coupling parameter ϵ is left open for tuning.

The above equations are differenciated and the system is solved using the second-order Runge–Kutta scheme with a time stepsize Δt=0.001. Initialized with random numbers, the state is integrated forward for N= 50,000 steps (t=50). Discard the initial 10,000 steps and form the 9 time series with 40,000 data points.

**Figure 2:**
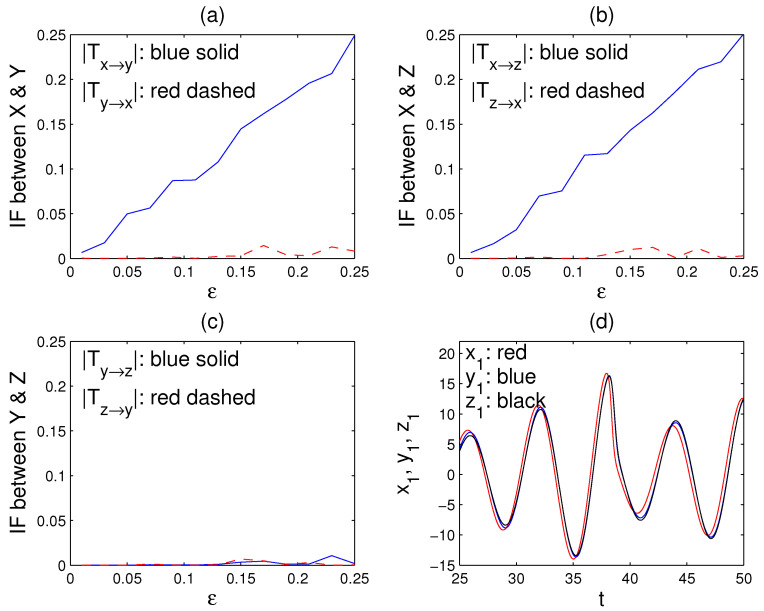
The information flows among the oscillators *X*, *Y*, and *Z* (in nats/unit time) versus the coupling strength ϵ: (**a**) $|T_{X\to Y}|$ (blue) and $|T_{Y\to X}|$ (red); (**b**) $|T_{X\to Z}|$ (blue) and $|T_{Z\to X}|$ (red); (**c**) $|T_{Y\to Z}|$ (blue) and $|T_{Y\to Z}|$ (red). (**d**) The series of $X_{1}$, $Y_{1}$, and $Z_{1}$ on a time interval when the coupling parameter ϵ=0.25. (Note, in solving for (X,Y,Z), the initial conditions are randomly chosen, some of which may happen to make a highly singular matrix and hence cause large errors. In that case, simply re-run the program.)

The oscillators are highly chaotic. As ϵ increases, the three oscillators gradually become in pace. They become almost synchronized after ϵ>0.15. Shown in Figure 2d is an episode of the synchronization for ϵ=0.25.

We now apply (14) to compute the information flows among *X*, *Y*, and *Z*. Since this is a deterministic chaos problem, choose k=2 in (7) and (14). Following [33], the series $\left\{x_{1}\left(n\right)\right\}$, $\left\{y_{1}\left(n\right)\right\}$, and $\left\{z_{1}\left(n\right)\right\}$ are used to represent the three oscillators. Shown in Figure 2a–c are dependencies of the computed information flows on the coupling strength ϵ. Clearly, among the six information flows, only $T_{X\to Y}$ and $T_{X\to Z}$ are significant, indicating (1) that the causal relation between *X* and *Y* is unidirectionally from *X* to *Y*, (2) that the causality between *X* and *Z* is also one-way, i.e., from *X* to *Z*, and, mostly importantly (3) that no direct causality exists between *Y* and *Z*, although they are highly correlated (c.f. Figure 2d). So, here the confoundingness is not at all an issue.

After ϵ exceeds 0.15, the systems begin to synchronize (see [33]), and it is impossible to infer the causal relation using traditional methods. This is understandable, as the series gradually approach toward one series. Here, however, even with ϵ>0.15, i.e., even after the series are almost synchronized, in this framework, the inference still performs remarkably well, as clearly seen in Figure 2a–c. This attests to the power of the information flow-based causal inference technique, which is concise and very easy to implement.

## 6. Conclusions

Recent years have seen a surge of interest in causality analysis. This study introduced a line of work starting some 16 years ago which has gone almost unnoticed, and implemented the state-of-the-art theory [16] into an easy-to-use algorithm. Particularly, this study extended the bivariate time series analysis of [18] to the long-due multivariate time series causal inference.

In a multivariate stochastic system, the information flow from one component to another proves to be (2). When only time series are available, it can be estimated using (14) under a linear assumption. Ideally if it is not zero, then there exists causality between the components, but practically statistical significance needs to be tested. These have been easily implemented as an algorithm for use.

More than just finding the information flows, hence the causalities, among the units (as in [18]), we have also estimated the influence of a unit to itself. This results in autocorrelation, which becomes an issue in some causal inferences. The consequence is that, in a causal graph, those nodes which are self loops (cycles of length 1) can be easily identified. Also different from previous studies, in a unified treatment, the role of noise has been quantified along with the causality analysis. This quantity has an easy physical interpretation, namely, the ratio of noise to signal. Besides, the obtained causalities can be normalized to measure the importance of the respective parental nodes.

The above very concise and transparent formulas have been applied to examine two problems in extreme situations: (1) a network of multivariate processes with heavy noise (stochastic perturbation amplitude 100 times the signal amplitude); (2) a network with nearly synchronized oscillators. Besides, confounding processes exist in both causal graphs. Case (1) is made of vector autoregressive processes. By applying the algorithm, the causal graph is accurately recovered in a very easy and efficient way. In particular, the confounding processes have been accurately clarified.

Note Granger causality testing works well in case (1). Nonetheless, the simplicity of the Formula (14) allows for an increase of performance by at least two orders.

In case (2), the network is formed with three chaotic Rössler oscillators. When the coupling coefficient exceeds a threshold, synchronization occurs. However, even with the almost completely synchronized time series, the information flow approach still performs remarkably well, with the causalities accurately inferred, and the causal graph accurately reconstructed. In particular, the one-way causalities between the master–slave systems have been recovered. Moreover, it is accurately shown that the two highly correlated, almost identical series due to the confounder are not causally linked.

It should be mentioned that, in arriving at the concise formula for causal inference, an assumption of linearity has been invoked. For some nonlinear problems, the inference may not be precisely as expected. For example, in Figure 2a,b, the red dashed lines are supposed to be zero, but here they are not. However, qualitatively, the inference is still good, as the one-way causality is clearly seen. Such success has already been evidenced in the bivariate case of [18], where a highly nonlinear problem defying classical approaches is examined. Nonetheless, the power of the information flow-based causality analysis will not be fully realized until the linear assumption is relaxed. To generalize to the fully nonlinear case is hence the goal of future work.

## Data Availability

Not Applicable.
